# Relationship between key continuous glucose monitoring-derived metrics and specific cognitive domains in patients with type 2 diabetes mellitus

**DOI:** 10.1186/s12883-023-03242-2

**Published:** 2023-05-20

**Authors:** Shanshan Dong, Lina Wang, Chenxu Zhao, Rui Zhang, Zhaoyu Gao, Lei Jiang, Yingying Guo, Huimin Zhou, Shunjiang Xu

**Affiliations:** 1 Department of Endocrinology and Metabolism, The First Hospital of Hebei Medical University, No.89, Donggang Road, Shijiazhuang, 050031 P. R. China; 2 Central Laboratory, The First Hospital of Hebei Medical University, Shijiazhuang, 050031 P. R. China; 3 Hebei International Joint Research Center for Brain Science, Shijiazhuang, 050031 P. R. China; 4 Hebei Key Laboratory of Brain Science and Psychiatric-Psychologic Disease, Shijiazhuang, 050031 P. R. China

**Keywords:** Time below range (TBR), Time in range (TIR), Time above range (TAR), Type 2 diabetes mellitus (T2DM), Cognitive impairment

## Abstract

**Background:**

Continuous glucose monitoring (CGM)-derived time in range (TIR) is closely associated with micro- and macrovascular complications in type 2 diabetes mellitus (T2DM). This study was performed to investigate the relationship between key CGM-derived metrics and specific cognitive domains in patients with T2DM.

**Methods:**

Outpatients with T2DM who were otherwise healthy were recruited for this study. A battery of neuropsychological tests was performed to evaluate cognitive function, including memory, executive functioning, visuospatial ability, attention, and language. Participants wore a blinded flash continuous glucose monitoring (FGM) system for 72 h. The key FGM-derived metrics were calculated, including TIR, time below range (TBR), time above range (TAR), glucose coefficient of variation (CV), and mean amplitude of glycemic excursions (MAGE). Furthermore, the glycemia risk index (GRI) was also calculated by the GRI formula. Binary logistic regression was used to assess risk factors for TBR, and we further analysed the associations between neuropsychological test results and key FGM-derived metrics with multiple linear regressions.

**Results:**

A total of 96 outpatients with T2DM were recruited for this study, with 45.8% experiencing hypoglycemia (TBR^< 3.9 mmol/L^). Spearman analysis results revealed that a higher TBR^< 3.9 mmol/L^ was correlated with worse performance on the Trail Making Test A (TMTA), Clock Drawing Test (CDT), and cued recall scores (*P* < 0.05). Logistic regression analysis results indicated that the TMTA (OR = 1.010, *P* = 0.036) and CDT (OR = 0.429, *P* = 0.016) scores were significant factors influencing the occurrence of TBR^< 3.9 mmol/L^. Multiple linear regressions further demonstrated that TBR^< 3.9 mmol/L^ (β = -0.214, *P* = 0.033), TAR^> 13.9 mmol/L^ (β = -0.216, *P* = 0.030) and TAR^10.1–13.9 mmol/L^ (β = 0.206, *P* = 0.042) were significantly correlated with cued recall scores after adjusting for confounding factors. However, TIR, GRI, CV and MAGE showed no significant correlation with the results of neuropsychological tests (*P* > 0.05).

**Conclusions:**

A higher TBR^< 3.9 mmol/L^ and TAR^> 13.9 mmol/L^ were associated with worse cognitive functions (memory, visuospatial ability, and executive functioning). Conversely, a higher TAR of 10.1–13.9 mmol/L was associated with better memory performance in memory tasks.

**Supplementary Information:**

The online version contains supplementary material available at 10.1186/s12883-023-03242-2.

## Background

According to the International Diabetes Federation 2021 report, the number of people affected with diabetes worldwide is 536.6 million, which is estimated to reach 783.2 million in 2045 [1]. Over 90% of people with diabetes have type 2 diabetes mellitus (T2DM) [2, 3]. People with T2DM are at risk of life-threatening acute and chronic complications, especially central nervous system damage, which can cause functional and structural changes in the brain that lead to cognitive impairment and behavioral deficits [4]. Chronic hyperglycemia, recurrent hypoglycemic episodes, and glycemic excursions have been implicated as potential causative factors of cognitive impairment [5–7]. Therefore, continuous glucose monitoring (CGM) has evolved as an essential part of diabetes management for diabetic patients with cognitive impairment. With the development of CGM technology, flash continuous glucose monitoring (FGM) systems have increased in popularity in recent years because of the highly detailed information and enhanced accuracy they provide. The key FGM-derived metrics enable quantitative evaluation of the quality of short-term glycemic control, including time in range (TIR), time below range (TBR), time above range (TAR), glucose coefficient of variation (CV), and mean amplitude of glycemic excursions (MAGE) [8]. However, as a single metric, TIR does not indicate whether the out-of-range readings are too low or too high; therefore, several researchers have proposed a new evaluation index of blood glucose the glycemia risk index (GRI) [9]. The GRI is based on weighted combinations of TBR (the hypoglycemia component) and TAR (the hyperglycemia component) [9]. As such, we believe that key FGM-derived metrics and GRI could provide more direct, comprehensive, and complete information on blood glucose levels.

Human cognitive function is a complex construct composed of seven key cognitive domains: learning and memory, visuospatial ability, executive functioning, language, complex attention, perceptual-motor function, and social cognition [10]. Cognitive impairment refers to the impairment of one or more of these cognitive domains [10]. Recently, it was shown that glucose variability is associated with disruption of executive functioning, attention and language ability [11]. Glucose variability increases the production of reactive oxygen species (ROS) [12]. Overproduction of ROS can cause oxidative stress, which leads to neuronal damage and apoptosis [13]. Thus, glucose variability induces the activation of oxidative stress, which may be a major contributor to the development of cognitive impairment [12–14]. Previous studies have shown that TIR, TBR, MAGE, and severe hypoglycemia are associated with cognitive impairment [11, 14–17]. However, there is a lack of understanding regarding the specific relationships between TBR/TAR/TIR/GRI and different cognitive domains in patients with T2DM. In this study, we conducted a cross-sectional analysis to explore the relationship between key FGM-derived metrics and specific cognitive domains in patients with T2DM to prevent the occurrence and development of cognitive impairment.

## Research design and methods

### Participants

Outpatients with T2DM who were otherwise healthy were recruited from the First Hospital of Hebei Medical University. This study recruited outpatients who met the following inclusion criteria: (1) age between 40 and 80 years old; (2) patients with T2DM who met the 1999 World Health Organization (WHO) diagnostic criteria for diabetes mellitus [18] and had been on a stable glucose-lowering regimen for the past 3 months. The exclusion criteria included (1) diabetic ketoacidosis, hyperglycemic-hyperosmolar state or severe hypoglycemia and recurrent episodes of hypoglycemia within the past 3 months, thyropathy, parathyropathy or other endocrinopathies; (2) major medical illness, such as severe anemia, serious heart disease, liver, and kidney dysfunction or other systemic diseases; (3) acute infection; (4) malignant tumors and autoimmune diseases; (5) severe neurological or psychiatric diseases, alcoholism or abuse of psychotropic medicines; and (6) large infarcts, infection, tumor, or multifocal gray matter and/or white matter lesions observed on magnetic resonance imaging (MRI).

### Clinical and biochemical measurements

Patient information on age, sex, diabetes duration, education, and prescription medications, such as glucose-lowering drugs, was collected by doctors. Each patient underwent a physical examination that included measurements of height, weight, and blood pressure. Blood samples were drawn by an experienced nurse after a 10-h overnight fast. Total cholesterol, triglycerides, high-density lipoprotein (HDL), and low-density lipoprotein (LDL) were determined by applying standard enzymatic methods using a biochemical analyzer (US Beckman AU5800 automatic chemistry analyzer, Beckman Coulter, America). Fasting plasma glucose (FPG) levels were assayed by using the glucose hexokinase method. Hemoglobin A1c (HbA1c) was measured by using high-performance liquid chromatography with a Hemoglobin A1c analyzer (Afinion 2 HbA1c, Abbott Diagnostics Technologies AS, Norway).

### Neuropsychological assessment

Several neuropsychological tests, including the Mini-Mental State Examination (MMSE) [19], Montreal Cognitive Assessment (MoCA) [20], Digit Span Test forward (DST forward) and backward (DST backward) [21], Trail Making Test A and B (TMTA and TMTB) [22], Boston Naming Test (BNT) [23], self-assessment of depression [24], Clock Drawing Test (CDT) [25, 26], Verbal Fluency Test (VFT) [27] and Auditory Verbal Learning Test (AVLT) [28], were performed to evaluate cognitive function, such as memory, executive functioning, visuospatial ability, attention and language. The AVLT included immediate memory total scores and scores on 20-min delayed recall, cued recall, and long-delayed recognition.

### FGM parameters

Each patient wore a blinded FGM system (FreeStyle Libre H; Abbott Diabetes Care, Witney, UK) for 72 h [29, 30]. The glucose data were automatically recorded and saved every 15 min, and finger blood correction was exempted. The sensor of the FGM system was inserted on Day 0. After 72 h of monitoring, the data were downloaded to a computer and analysed. Key FGM-derived metrics were calculated. These metrics included the following: (1) TIR: % of readings and time in the range of 3.9–10.0 mmol/L. (2) TBR: % of readings and time at < 3.9 mmol/L (TBR^< 3.9 mmol/L^). According to the value of TBR^< 3.9 mmol/L^, the participants were divided into two groups based on absence or presence of hypoglycemia (with or without hypoglycemia). Furthermore, the TBR values were classified as level 1 hypoglycemia (% of readings and time in the range of 3.0-3.8 mmol/L (TBR^3.0–3.8 mmol/L^)) and level 2 hypoglycemia (% of readings and time at < 3.0 mmol/L (TBR^< 3.0 mmol/L^)). Nocturnal asymptomatic hypoglycemia (NAH) was defined as T2DM patients with glucose levels < 3.9 mmol/L without the typical symptoms of hypoglycemia occurring between 0:00 am and 6:00 am [29]. (3) TAR: % of readings and time at > 10.0 mmol/L. The TAR values were further classified as level 1 hyperglycemia (% of readings and time in the range of 10.1–13.9 mmol/L (TAR^10.1–13.9 mmol/L^)) and level 2 hyperglycemia (% of readings and time at > 13.9 mmol/L (TAR^> 13.9 mmol/L^)) [8]. (4) CV and MAGE. GRI was calculated through the GRI formula. ‘.

### Statistical analysis

Statistical analyses were carried out with the IBM SPSS 25.0 software package (IBM Corp., Armonk, N.Y., USA). Continuous variables with a normal distribution are presented as the mean ± SD, and those not conforming to a normal distribution are expressed as the interquartile range [M (QL, QU)]. For continuous variables with normal or skewed distributions, Student’s t test, one-way ANOVA or the Mann‒Whitney U test were used for comparisons between groups. The χ2-test was used for categorical variables. Spearman analysis was used to analyse the relationships between glucose metrics and neuropsychological test results. Binary logistic regression was used to analyse the associations between TBR and neuropsychological test results. Multilinear regression analysis was used to analyse the correlations between neuropsychological test results and FPG, key FGM-derived metrics and GRI.

## Results

### Characteristics of the study participants

A total of 96 outpatients with T2DM were recruited for this study. Participant characteristics are shown in Table 1. The mean age of the 96 patients was 61.40 ± 7.84 years, 59.38% (n = 57) of the patients were male and 40.62% (n = 39) were female, the duration of diabetes was 13.05 ± 7.81 years, HbA1c was 7.85% (7.20%, 8.70%), and CV% was 26.50% (23.65%, 33.00%). A total of 45.8% of patients with T2DM had hypoglycemia (TBR^< 3.9 mmol/L^). Among them, 23 patients (23.96%) had NAH. There were no significant differences in sex, age, duration of disease, education, blood pressure, BMI or blood lipid levels between the groups with and without hypoglycemia (*P* > 0.05). Compared with T2DM patients without hypoglycemia, T2DM patients with hypoglycemia showed increased TIR and CV values (*P* < 0.01) and lower HbA1c, FPG, TAR^10.1–13.9 mmol/L^, TAR^> 13.9 mmol/L^, and mean glucose (MG) values (all *P* < 0.01). T2DM patients with hypoglycemia exhibited significantly worse performance on the TMTA and CDT (all *P* < 0.05) (Table 2). Moreover, compared with T2DM patients without hypoglycemia, T2DM patients with level 1 hypoglycemia (TBR^3.0–3.8 mmol/L^) exhibited significantly worse performance on the TMTA and CDT (all *P* < 0.05), as shown in Supplementary Table S1.

**Table 1:** Characteristics of study participants

Variable	Total (*n* = 96)	Without hypoglycemia (*n* = 52)	With hypoglycemia (*n* = 44)	*P* values
Male, n (%)	57 (59.38)	27 (51.92)	30 (68.18)	0.106
Age (years)	61.40 ± 7.84	61.06 ± 8.57	61.80 ± 6.96	0.648
**Education**: junior college degree or above, n (%)	41 (42.71)	20 (38.46)	21 (47.73)	0.087
Diabetes duration (years)	13.05 ± 7.81	13.00 ± 7.69	13.11 ± 8.03	0.944
SBP (mmHg)	133.89 ± 13.88	133.88 ± 14.37	133.89 ± 13.44	1.000
DBP (mmHg)	80.68 ± 9.07	81.15 ± 8.61	80.11 ± 9.66	0.578
BMI (kg/m^2^)	26.11 ± 3.91	26.64 ± 4.15	25.48 ± 3.56	0.150
Total cholesterol (mmol/L)	4.84 ± 1.09	4.90 ± 1.12	4.76 ± 1.06	0.510
Triglycerides (mmol/L)	1.39 (0.98, 2.19)	1.68 (0.93, 2.46)	1.31 (1.01, 1.85)	0.184
HDL (mmol/L)	1.18 (1.03, 1.39)	1.19 (1.04, 1.40)	1.16 (1.01, 1.32)	0.476
LDL (mmol/L)	3.00 ± 0.79	3.03 ± 0.76	2.97 ± 0.83	0.753
HbA1c (%)	7.85 (7.20, 8.70)	8.15 (7.43, 9.08)	7.45 (6.70, 8.18)	< 0.001
FPG (mmol/L)	8.50 (7.13, 10.47)	8.83 (7.69, 11.58)	7.91 (6.21, 9.44)	0.005
TIR (%)	61.66 ± 27.76	52.34 ± 31.25	72.68 ± 17.73	< 0.001
TAR^10.1–13.9 mmol/L^ (%)	20.50 (11.26, 33.27)	29.30 (16.75, 41.75)	16.08 (6.00, 21.05)	< 0.001
TAR^> 13.9 mmol/L^ (%)	2.91 (0.00, 18.00)	8.60 (0.09, 28.86)	1.05 (0.00, 6.81)	0.002
SD (mmol/L)	2.60 ± 0.84	2.59 ± 0.73	2.61 ± 0.96	0.883
GRI	34.32 (20.00, 64.60)	39.75 (18.18, 83.30)	29.38(20.98, 47.03)	0.158
MG (mmol/L)	9.27 ± 2.76	10.50 ± 2.88	7.81 ± 1.71	< 0.001
CV (%)	26.50 (23.65, 33.00)	24.77 (21.61, 27.02)	32.15 (26.85, 37.50)	< 0.001
MAGE (mmol/L)	5.66 (4.21, 7.00)	5.80 (4.36, 6.81)	5.24 (3.98, 7.66)	0.903
**Use antidiabetes agents**				
Insulin, n (%)	47 (48.96)	27 (51.92)	20 (45.45)	0.528
Sulfonylurea, n (%)	19 (19.79)	11 (21.15)	8 (18.18)	0.716
Biguanides, n (%)	67 (69.79)	38 (73.08)	29 (65.91)	0.446
α-glucosidase inhibitors, n (%)	51 (53.13)	27 (51.92)	24 (54.55)	0.798
Dipeptidyl peptidase 4 inhibitors, n (%)	10 (10.42)	6 (11.54)	4 (9.09)	0.696
Glucagon-like peptide 1 receptor agonists, n (%)	10 (10.42)	4 (7.69)	6 (13.64)	0.342
Sodium–glucose cotransporter 2 inhibitors, n (%)	5 (5.21)	3 (5.77)	2 (4.55)	0.788

Data are presented as means ± standard deviations or medians (interquartile ranges) for continuous variables, and numbers (percentages) for categorical variables. A two-tailed value of *P* < 0.05 was considered as statistically significant.

SBP, systolic blood pressure; DBP, diastolic blood pressure; BMI, body mass index; HDL, high-density lipoprotein; LDL, low-density lipoprotein; HbA1c, hemoglobin A1c; FPG, fasting plasma glucose; TIR, time in range; TAR, time above range; SD, standard deviation; GRI, glycemia risk index; MG, mean glucose; CV, coefficient of variation; MAGE, mean amplitude of glycemic excursions

**Table 2:** Neuropsychological test information in all patients

Variable	Without hypoglycemia (*n* = 52)	With hypoglycemia (*n* = 44)	*P* values
MMSE	28.00 (27.00, 29.00)	28.00 (26.00, 29.00)	0.418
MoCA	23.37 ± 2.69	22.70 ± 3.35	0.286
Immediate memory total scores	23.81 ± 5.07	22.34 ± 5.16	0.165
20-min delayed recall	8.33 ± 2.20	7.64 ± 3.09	0.206
Cued recall	10.50 (9.00, 11.75)	10.00 (8.00, 11.00)	0.118
Long delayed recognition	12.00 (11.00, 13.75)	12.00 (10.00, 14.00)	0.965
DST forward	7.00 (6.00, 8.00)	6.00 (6.00, 8.00)	0.114
DST backward	4.00 (4.00, 5.00)	4.00 (3.00, 5.00)	0.503
TMTA	38.43 (32.43, 60.25)	62.79 (38.16, 150.00)	0.010
TMTB	88.00 (57.93, 300.00)	300.00 (72.37, 300.00)	0.053
BNT	23.50 (22.00, 27.00)	24.00 (21.00, 26.00)	0.799
CDT	3.00 (2.00, 3.00)	2.00 (1.25, 3.00)	0.028
VFT	18.00 (15.00, 20.00)	17.00 (14.00, 21.00)	0.507
Depression scale	51.88 (47.50, 57.50)	52.50 (47.50, 56.25)	0.997
Negative emotion	86.50 (76.00, 95.00)	87.50 (76.75, 92.75)	0.953

Data are presented as means ± standard deviations or medians (interquartile ranges) for continuous variables. A two-tailed value of *P* < 0.05 was considered as statistically significant.

MMSE, Mini-Mental State Examination; MoCA, Montreal Cognitive Assessment; DST, Digit Span Test; TMT, Trail Making Test; BNT, Boston Naming Test; CDT, Clock Drawing Test; VFT, Verbal Fluency Test

### Associations between TBR^< 3.9 mmol/L^ and neuropsychological test results

**Fig. 1:**
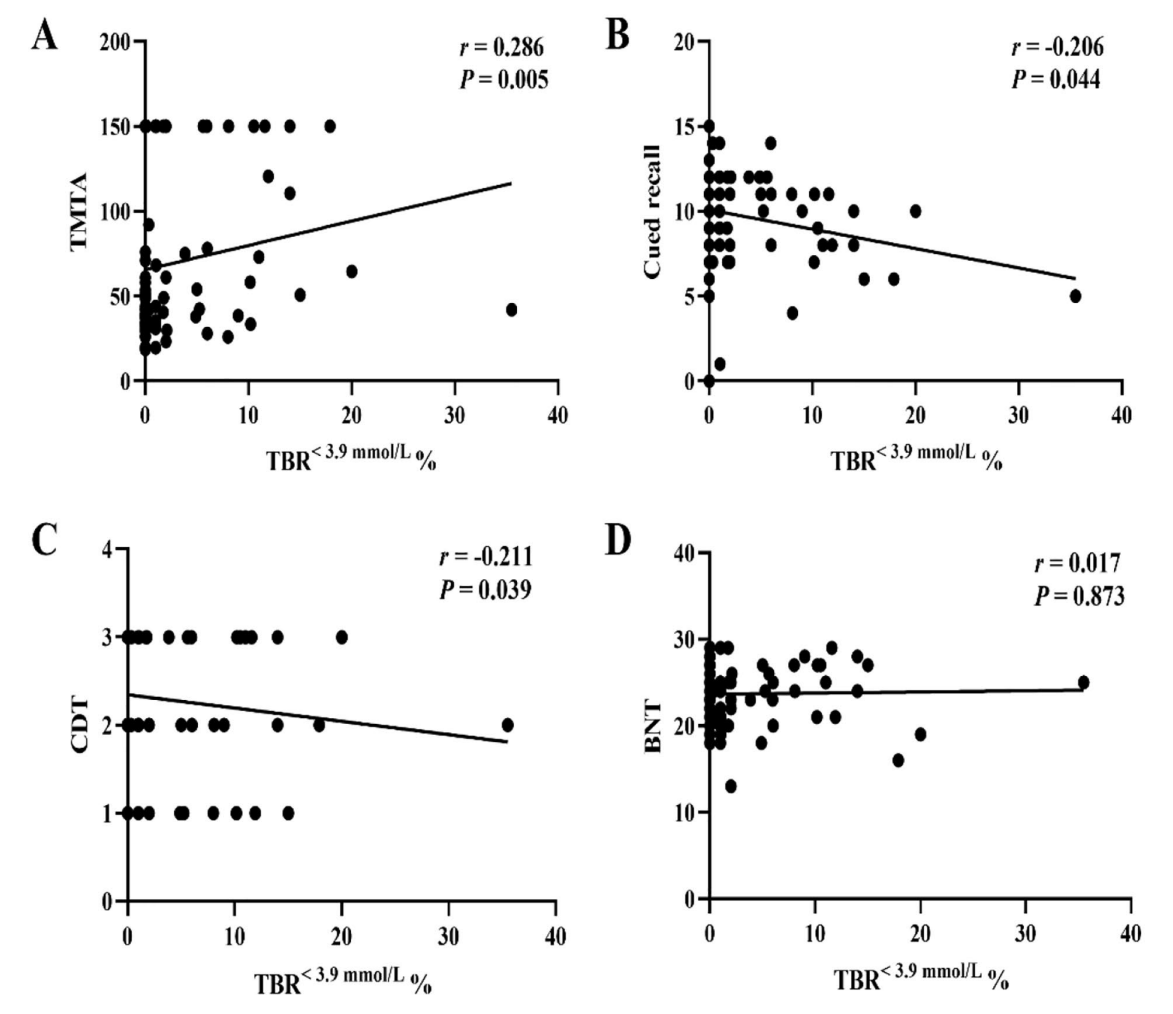
Correlation of TBR^< 3.9 mmol/L^ and neuropsychological test results. **A**, Correlation between TBR^< 3.9 mmol/L^ and TMTA scores. **B**, Correlation between TBR^< 3.9 mmol/L^ and cued recall scores. **C**, Correlation between TBR^< 3.9 mmol/L^ and CDT scores. **D**, Correlation between TBR^< 3.9 mmol/L^ and BNT scores. TMTA, Trail Making Test A; BNT, Boston Naming Test; CDT, Clock Drawing Test; TBR, time below range

**Fig. 2:**
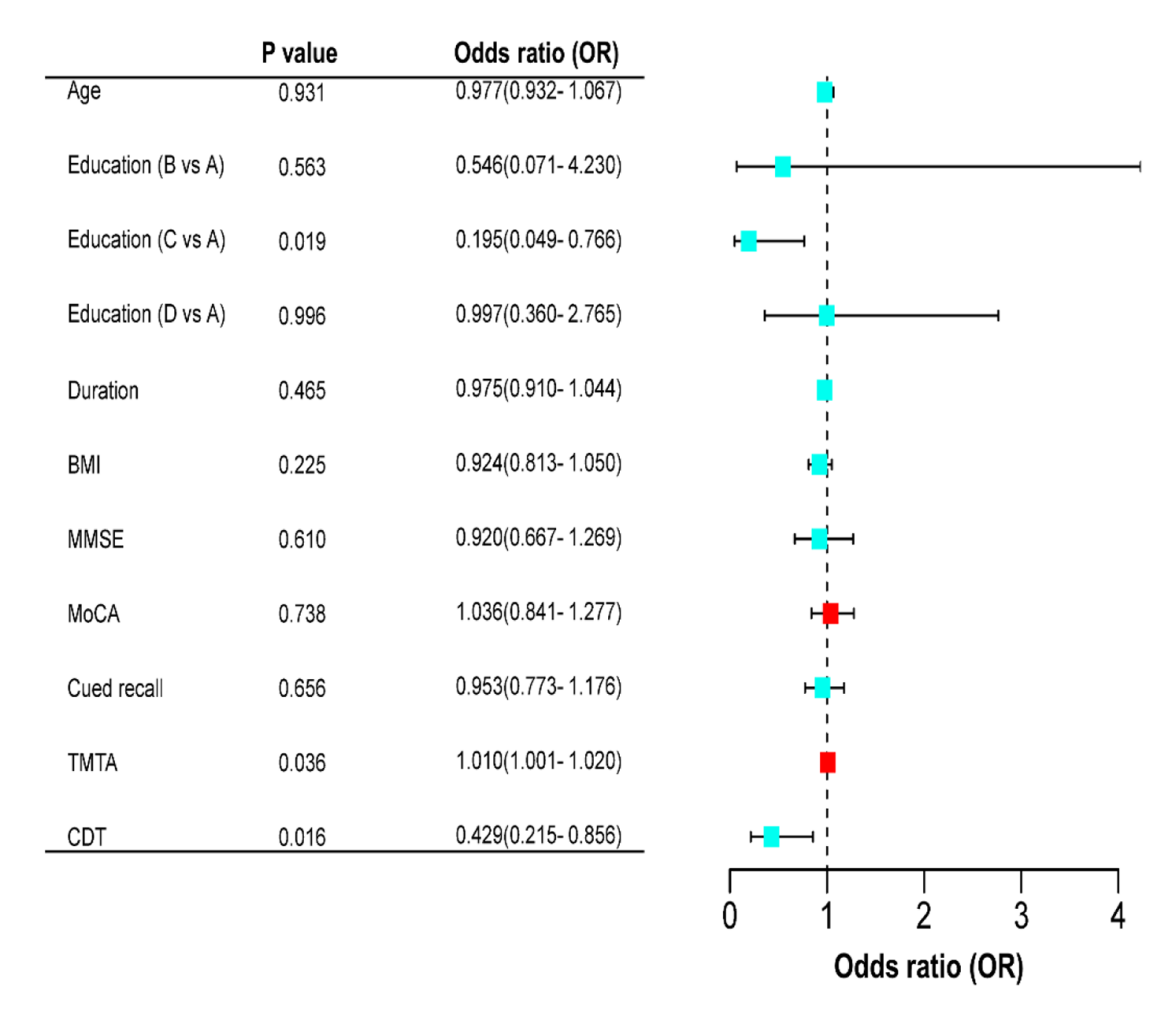
Logistic regression analysis of risk factors for the development of T2DM combined with TBR^< 3.9 mmol/L^. MMSE, Mini-Mental State Examination; MoCA, Montreal Cognitive Assessment; TMTA, Trail Making Test A; CDT, Clock Drawing Test; TBR, time below range. Education, A: primary school; B: junior middle school; C: senior high school; D: junior college degree

The correlation analysis of TBR^< 3.9 mmol/L^ with neuropsychological test results was performed with Spearman’s analysis. TBR^< 3.9 mmol/L^ was positively correlated with TMTA scores (*P* < 0.01) and negatively correlated with cued recall and CDT scores (*P* < 0.05) (Fig. 1). Binary logistic regression analysis was carried out to further analyse the association between TBR and neuropsychological test results. The binary dependent variables were TBR^< 3.9 mmol/L^ (T2DM patients with glucose levels < 3.9 mmol/L were coded as 1, and T2DM patients without glucose levels < 3.9 mmol/L were coded as 0), and the independent variables included age, duration of disease, education, BMI, and neuropsychological test results (scores on MMSE, MoCA, CDT, TMTA and cued recall). The results revealed that TMTA (OR = 1.010, *P* = 0.036) and CDT (OR = 0.429, *P* = 0.016) scores were independent influencing factors for the occurrence of TBR^< 3.9 mmol/L^ (Fig. 2). After adjusting for age, sex, diabetes duration, education, and BMI, significant associations existed between CDT scores (OR = 0.379, *P* = 0.008) and TBR^< 3.9 mmol/L^. There was still a significant correlation after further adjustment for scores on the TMTA (*P* < 0.05) (Table 3).

**Table 3:** Association between CDT scores and TBR^< 3.9 mmol/L^ after controlling for confounding factors

Models	TBR^< 3.9 mmol/L^		
	OR	95% CI	*P* values
**Model 1**			
CDT	0.379	(0.185–0.778)	0.008
**Model 2**			
CDT	0.385	(0.186–0.800)	0.011
TMTA	1.010	(0.999–1.021)	0.071

Model 1 was adjusted for age, sex, duration of disease, education and BMI.

Model 2 includes all variables in Model 1 plus TMTA.

TMTA, Trail Making Test A; CDT, Clock Drawing Test

### Associations between FPG and neuropsychological test results

**Fig. 3:**
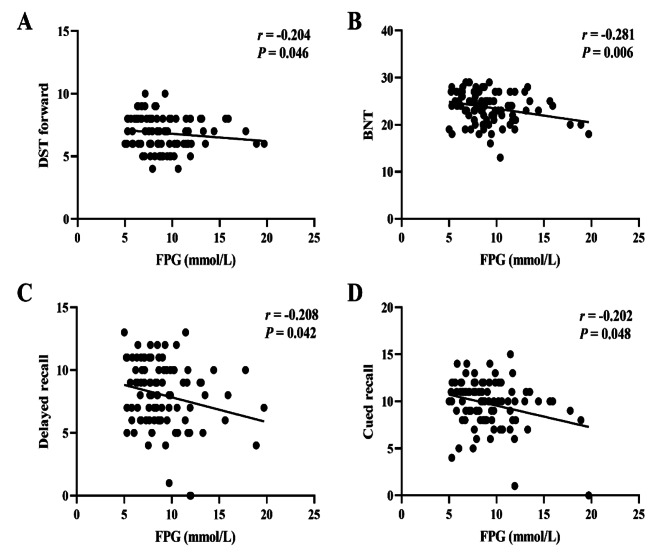
Correlation of FPG and neuropsychological test results. **A**, Correlation between FPG and DST forward scores. **B**, Correlation between FPG and BNT scores. **C**, Correlation between FPG and delayed recall scores. **D**, Correlation between FPG and cued recall scores. DST, Digit Span Test; BNT, Boston Naming Test; FPG, fasting plasma glucose

FPG was negatively correlated with scores on DST forward, BNT, delayed recall, and cued recall (*P* < 0.01 or *P* < 0.05) (Fig. 3).

### Associations between cued recall scores and key FGM-derived metrics

After adjusting for age, sex, diabetes duration, education, and BMI, FPG (β = -0.307, *P* = 0.003), TBR^< 3.9 mmol/L^ (β = -0.214, *P* = 0.033), TAR^> 13.9 mmol/L^ (β = -0.216, *P* = 0.030) and TAR^10.1–13.9 mmol/L^ (β = 0.206, *P* = 0.042) were significantly correlated with cued recall scores, and there was still a significant correlation after adjustment for depression scale scores (*P* < 0.05). After adjusting for FPG, the correlation of TAR^> 13.9 mmol/L^ (β = -0.031, *P* = 0.818) disappeared, but TBR^< 3.9 mmol/L^ (β = -0.327, *P* = 0.001) and TAR^10.1–13.9 mmol/L^ (β = 0.343, *P* = 0.001) were even more significantly correlated with cued recall scores (Table 4).

**Table 4:** Association between cued recall scores and FPG/TBR/TAR/TIR/GRI after controlling for confounding factors

	β	95% CI	*P* values
**Model 1**			
FPG	-0.307	(-0.441 - -0.092)	0.003
**Model 2**			
TBR^< 3.9 mmol/L^ (%)	-0.214	(-0.186 - -0.008)	0.033
TAR^> 13.9 mmol/L^ (%)	-0.216	(-0.053 - -0.003)	0.030
TAR^10.1–13.9 mmol/L^ (%)	0.206	(0.001–0.067)	0.042
TIR (%)	0.086	(-0.011–0.026)	0.399
GRI	-0.154	(-0.030–0.004)	0.129
**Model 3**			
TBR^< 3.9 mmol/L^ (%)	-0.327	(-0.234 - -0.062)	0.001
TAR^> 13.9 mmol/L^ (%)	-0.031	(-0.038–0.030)	0.818
TAR^10.1–13.9 mmol/L^ (%)	0.343	(0.025–0.089)	0.001
**Model 4**			
TBR^< 3.9 mmol/L^ (%)	-0.212	(-0.186 - -0.006)	0.036
TAR^> 13.9 mmol/L^ (%)	-0.218	(-0.054 - -0.003)	0.030
TAR^10.1–13.9 mmol/L^ (%)	0.203	(0.001–0.067)	0.046

Model 1 was adjusted for age, sex, duration of disease, education and BMI.

Model 2 was adjusted for age, sex, duration of disease, education and BMI.

Model 3 includes all variables in model 2 plus FPG.

Model 4 includes all variables in model 2 plus depression scale.

FPG, fasting plasma glucose; TIR, time in range; TAR, time above range; TBR, time below range

GRI, glycemia risk index

### Associations between TIR/GRI/CV/MAGE and neuropsychological test results

Multiple linear regression was used to investigate the associations between TIR/GRI/CV/MAGE and neuropsychological test results, and the results revealed that TIR, GRI, CV and MAGE had no correlation with neuropsychological test results (*P* > 0.05) (Supplementary Table S2).

All of the participants were stratified according to tertiles of TIR (TIR < 57%; 57% ≤ TIR < 79%; TIR ≥ 79%). In patients with the same incidence of TBR^< 3.9 mmol/L^, compared with T2DM patients with 57% ≤ TIR < 79%, T2DM patients with TIR ≥ 79% exhibited significantly better performance on the BNT (*P* = 0.020), and there was no difference in other neuropsychological test results (*P* > 0.05) (Supplementary Table S3).

## Discussion

The present study demonstrated that a higher TBR^< 3.9 mmol/L^ was associated with worse cognitive performance, especially with memory, visuospatial ability and executive functioning. A higher TAR^> 13.9 mmol/L^ was associated with worse memory performance. Conversely, a higher TAR^10.1–13.9 mmol/L^ was associated with better memory performance in outpatients with T2DM.

Previous studies have indicated that both severe hypoglycemia and recurrent episodes of hypoglycemia were related to cognitive impairment in patients with T2DM [31, 32]. In another study, it was demonstrated that TBR was negatively correlated with MoCA scores [15]. However, our study found no correlation between TBR^< 3.9 mmol/L^ and MoCA scores. This disparity in results may be due to differences in the study population, as the patients in our study were relatively younger (mean age: 61.40 years) than those in the previous study (mean age: 70.35 years) [15]. Several studies reported that diabetes-associated cognitive impairment could occur in adolescents and young adults with T2DM [33, 34], while other studies are inconsistent with this view [35].

Our study revealed that a higher TBR^< 3.9 mmol/L^ was correlated with worse performance on TMTA, CDT and cued recall. Therefore, TBR^< 3.9 mmol/L^ was found to be associated with cognitive impairment, particularly memory dysfunction, deficits in visuospatial ability, and impaired executive functioning in younger patients with T2DM, which is consistent with previous studies [17].

In addition, FGM provided an improved opportunity to capture NAH events, and we found that 23.96% of patients with T2DM experienced NAH. NAH was associated with neurological damage, which was consistent with previous studies [36]. The occurrence of NAH in patients might be related to disorders of rapid eye movement (REM) sleep phases and the suppression of counterregulatory hormone responses to hypoglycemia during REM sleep [37].

Furthermore, we conducted a comparison of TMTA and CDT performance between T2DM patients with level 1 hypoglycemia (TBR^3.0–3.8 mmol/L^) and T2DM patients without hypoglycemia. Our findings revealed that T2DM patients with level 1 hypoglycemia (TBR^3.0–3.8 mmol/L^) exhibited significantly worse performance on the TMTA and CDT, which indicates that nonsevere hypoglycemia is associated with cognitive impairment, specifically deficits in visuospatial ability and impaired executive functioning. However, we did not observe any significant association between TBR^< 3.9 mmol/L^ and cognitive performance in the global cognition, language, and attention domains.

Previous studies have demonstrated a bidirectional association between severe hypoglycemia and cognitive impairment in individuals with T2DM. Severe hypoglycemia can contribute to a decline in cognitive function, and cognitive impairment may increase the risk of severe hypoglycemia [38]. Our findings were consistent with numerous studies on T2DM, indicating that TBR^< 3.9 mmol/L^ was associated with disruption in memory, executive functioning, and visuospatial ability. Furthermore, we found that deficits in executive functioning and visuospatial ability may lead to the occurrence of TBR^< 3.9 mmol/L^, and the impairment of visuospatial ability was a more important factor for the occurrence of TBR^< 3.9 mmol/L^. Therefore, our study has shown a bidirectional association between TBR^< 3.9 mmol/L^ and cognitive impairment.

Hypoglycemic episodes can have detrimental effects on the brain, including neuronal death in the hippocampus and cerebral cortex, as well as increased consumption of alternate respiratory substrates, such as ketone bodies, glycogen and monocarboxylate, in the brain, leading to mitochondrial dysfunction and brain function damage [39, 40]. Moreover, patients with cognitive impairment may have a decreased ability to sense hypoglycemia, resulting in repeated episodes of hypoglycemia. In addition, patients with cognitive dysfunction may have a higher risk of hypoglycemia due to reduced capacity for self-care and the potential for overdose of glucose-lowering medications. This bidirectional association highlights the importance of managing hypoglycemia in patients with T2DM to prevent further cognitive decline.

In our study, we also investigated the association between hyperglycemia and cognitive performance using TAR and FPG as metrics of hyperglycemia. Previous studies have consistently shown a strong correlation between hyperglycemia and cognitive impairment [16, 41]. Our findings revealed that a higher TAR^10.1–13.9 mmol/L^ was associated with better memory performance. Even after adjusting for various confounding factors, TAR^10.1–13.9 mmol/L^ remained one of the main factors affecting memory. Therefore, we speculate that maintaining blood glucose levels within the range of 10.1–13.9 mmol/L may potentially slow the decline in memory and impede the occurrence and development of cognitive impairment. Notably, our study is the first to reveal that a higher TAR^10.1–13.9 mmol/L^ was associated with better cognitive function. Therefore, we recommended that glycemic control target values should be relaxed for T2DM patients with cognitive impairment.

However, it is important to note that another study demonstrated that a higher TAR^> 10.0 mmol/L^ was associated with a lower performance in executive functioning and working memory [16]. Our results are in line with these findings. We found that a higher TAR^> 13.9 mmol/L^ was correlated with worse memory performance in younger patients with T2DM. Additionally, previous studies have suggested that elevated FPG could increase the risk of dementia [42]. In our study, we observed that higher FPG levels were associated with worse memory, attention and language abilities. Even after adjusting for various confounding factors, FPG remained one of the main factors affecting memory.

As such, our findings suggest that a higher TAR^> 13.9 mmol/L^ and FPG levels were associated with worse cognitive function in younger patients with T2DM. The underlying mechanism for the association between very high-glucose hyperglycemia (TAR^> 13.9 mmol/L^) and cognition remains unknown. Acute high-glucose hyperglycemia may lead to cellular hypoxia and intracellular hyperosmosis in cerebral neurons, resulting in neuronal damage [43]. Chronic high-glucose hyperglycemia can cause the accumulation of advanced glycation end products, ROS, and proinflammatory cytokines that induce neuronal damage [44]. Additionally, T2DM patients with cognitive dysfunction may have a higher risk of hyperglycemia due to forgetting to take glucose-lowering medications, although hyperglycemia may also be caused by a combination of excessive food intake and lack of exercise.

TIR is closely associated with micro- and macrovascular complications and mortality in patients with T2DM [30, 45–47]. Several studies have reported that TIR is closely related to cognitive dysfunction [15, 16]. However, in contrast to a previous study, our findings did not reveal a relationship between TIR and global cognition or specific cognitive domains. The discrepancies in our findings may be attributable to various reasons, including the age and ethnicity of participants and the duration of FGM. Another potential factor contributing to the inconsistent outcome could be the high incidence of TBR^< 3.9 mmol/L^, which was observed in 45.8% of patients. Interestingly, patients with higher TIR had better language ability at the same incidence of TBR^< 3.9 mmol/L^. Therefore, when adjusting the glucose-lowering regimen for patients with T2DM, the DATAA (Download Data, Assess Safety, Time in Range, Areas to Improve, Action Plan) model should be adopted in interpreting the FGM data in elderly patients and those with cognitive impairment [48], and the first priority in this approach should be to reduce TBR to target levels, followed by addressing TIR. Moreover, implementing individualized glucose-lowering treatment regimens may lead to improvements in language ability and help prevent the occurrence and progression of cognitive impairment.

The GRI is a composite metric for evaluating glycemic control by utilizing the percentages of hyperglycemia and hypoglycemia based on CGM data. The lower the GRI is, the better the quality of glycemic control [9]. However, in our study, we did not observe an association between the GRI and specific cognitive domains, which may be attributed to the limitations of the GRI. The GRI cannot independently reflect the composition of hyperglycemia and hypoglycemia groups, and it may not predict the occurrence of cognitive impairment in patients with T2DM.

Several studies have suggested that cognitive dysfunction in patients with T2DM may be associated with glucose variability [11, 14]. However, a cross-sectional study in Japan found no association between CV and cognitive performance [16]. A CV% target ≤ 36% was defined as stable glucose, and a CV% target > 36% was defined as unstable glucose [49]. In the current study, we found that the CV% was 26.50% (23.65%, 33.00%), indicating stable blood glucose levels. As a result, there was no significant relationship between CV and global cognition or specific cognitive domains in the study population, which is consistent with the Japanese study. Previous studies have shown that MAGE correlated negatively with MMSE [14]. However, in our study, we did not find any association between MAGE and cognitive performance. One possible explanation for this discrepancy is the age difference between the two studies. The patients in our study were much younger (mean age: 61.40 years) than those in the other study (mean age: 78 years). Overall, our findings suggest that glucose variability may not significantly impact cognitive function in younger patients with T2DM.

This study had several limitations. The cross-sectional design did not allow us to explore the relationship between the key FGM-derived metrics and the development of cognitive impairment. The present study also had a small sample size, and expansion of the sample volume and follow-up observation studies on patients are necessary. In the future, we will collect more clinical samples in groups of different age stages, and participants will wear a blinded FGM system for 14 days.

## Conclusions

Through the study of the relationship between key FGM-derived metrics and specific cognitive domains, we found that a higher TBR^< 3.9 mmol/L^ was associated with worse cognition (memory dysfunction, deficits in visuospatial ability, and impaired executive functioning). At the same incidence of TBR^< 3.9 mmol/L^, the patients with a higher TIR exhibited better performance on language ability. In addition, we found that a higher TAR^10.1–13.9 mmol/L^ was associated with better memory performance, whereas a higher TAR^> 13.9 mmol/L^ was correlated with worse memory performance. Therefore, it is important to be relaxed when setting glycemic targets for T2DM patients with cognitive impairment, with a strong focus on reducing TBR^< 3.9 mmol/L^ and preventing high-glucose hyperglycemia (TAR^> 13.9 mmol/L^).

## Electronic supplementary material

Below is the link to the electronic supplementary material.

Supplementary Material 1

## Data Availability

The datasets used and/or analysed during the current study are available from the corresponding author on reasonable request.

## Abbreviations

CGM: Continuous glucose monitoring
T2DM: Type 2 diabetes mellitus
FGM: Flash continuous glucose monitoring
TIR: Time in range
TBR: Time below range
TAR: Time above range
GRI: Glycemia risk index
HDL: High-density lipoprotein
LDL: Low-density lipoprotein
FPG: Fasting plasma glucose
MMSE: Mini-Mental State Examination
MoCA: Montreal Cognitive Assessment
DST forward: Digit Span Test forward
DST backward: Digit Span Test backward
TMTA: Trail Making Test A
TMTB: Trail Making Test B
BNT: Boston Naming Test
CDT: Clock Drawing Test
VFT: Verbal Fluency Test
AVLT: Auditory Verbal Learning Test
NAH: Nocturnal asymptomatic hypoglycemia
REM: Rapid eye movement
SBP: Systolic blood pressure
DBP: Diastolic blood pressure
BMI: Body mass index
HbA1c: Hemoglobin A1c
SD: Standard deviation
MG: Mean glucose
CV: Coefficient of variation
MAGE: Mean amplitude of glycemic excursions
ROS: reactive oxygen species.
