# Supplementary material for: Relationship between key continuous glucose monitoring-derived metrics and specific cognitive domains in patients with type 2 diabetes mellitus
Source: BMC Neurol. 2023 May 20;23:200. doi: 10.1186/s12883-023-03242-2 (PMC10199495; doi:10.1186/s12883-023-03242-2)
Supplement: Supplementary file 1 — Supplementary Material 1 [file 12883_2023_3242_MOESM1_ESM.docx]

**Table S1** Neuropsychological test information by TBR^3.0-3.8 mmol/L^ group

| **Variable** | **Without hypoglycemia**  **(*n* = 52)** | **Level 1 hypoglycemia (TBR^3.0-3.8 mmol/L^)**  **(*n* = 31)** | **P values** |
| --- | --- | --- | --- |
| MMSE | 28.00 (27.00, 29.00) | 28.00 (26.00, 29.00) | 0.488 |
| MoCA | 23.37 ± 2.69 | 22.55 ± 3.31 | 0.223 |
| Immediate memory total score | 23.81 ± 5.07 | 22.74 ± 5.27 | 0.364 |
| 20-min delayed recall | 8.33 ± 2.20 | 7.58 ± 3.07 | 0.203 |
| Cued recall | 10.50 (9.00, 11.75) | 10.00 (8.00, 11.00) | 0.090 |
| Long delayed recognition | 12.00 (11.00, 13.75) | 12.00 (10.00, 14.00) | 0.816 |
| DST forward | 7.00 (6.00, 8.00) | 6.00 (6.00, 7.00) | 0.103 |
| DST backward | 4.00 (4.00, 5.00) | 4.00 (4.00, 5.00) | 0.847 |
| TMTA | 38.43 (32.43, 60.25) | 61.00 (35.46, 150.00) | 0.037 |
| TMTB | 88.00 (57.93, 300.00) | 300.00 (69.00, 300.00) | 0.146 |
| BNT | 23.50 (22.00, 27.00) | 24.00 (21.00, 26.00) | 0.715 |
| CDT | 3.00 (2.00, 3.00) | 2.00 (1.00, 3.00) | 0.010 |
| VFT | 18.00 ± 4.31 | 17.81 ± 5.50 | 0.859 |
| Depression scale | 52.68 ± 7.22 | 53.19 ± 5.67 | 0.738 |
| Negative emotion | 83.73 ± 13.71 | 83.97 ± 9.62 | 0.933 |

Data are presented as means ± standard deviations or medians (interquartile ranges) for continuous variables, and numbers (percentages) for categorical variables. A two-tailed value of *P* < 0.05 was considered as statistically significant.

MMSE, Mini-Mental State Examination; MoCA, Montreal Cognitive Assessment; DST, Digit Span Test; TMT, Trail Making Test; BNT, Boston Naming Test; CDT, Clock Drawing Test; VFT, Verbal Fluency Test; TBR, time below range.

**Table S2** Association between neuropsychological test information and TIR/GRI/CV/MAGE

|  | MMSE | MoCA | Immediate memory total score | 20-min delayed recall | Cued recall | Long delayed recognition | DST forward | DST backward |
| --- | --- | --- | --- | --- | --- | --- | --- | --- |
| TIR | -0.140 | -0.150 | -0.108 | 0.043 | 0.086 | 0.035 | -0.102 | -0.116 |
| GRI | 0.160 | 0.160 | 0.064 | -0.094 | -0.154 | -0.038 | 0.087 | 0.049 |
| CV | -0.064 | 0.019 | -0.137 | -0.036 | 0.012 | 0.125 | -0.040 | -0.125 |
| MAGE | -0.007 | 0.081 | 0.054 | -0.051 | 0.013 | 0.067 | 0.021 | -0.029 |

|  | TMTA | TMTB | BNT | CDT | VFT | Depression scale | Negative emotion |
| --- | --- | --- | --- | --- | --- | --- | --- |
| TIR | 0.182 | 0.059 | -0.046 | -0.116 | -0.083 | 0.052 | -0.093 |
| GRI | -0.179 | -0.061 | 0.089 | 0.065 | 0.084 | -0.028 | 0.032 |
| CV | 0.074 | 0.031 | 0.017 | -0.081 | 0.009 | -0.011 | -0.057 |
| MAGE | -0.141 | -0.047 | -0.037 | 0.034 | 0.012 | -0.028 | -0.011 |

Values are standardized regression coefficients (β). All the values have not statistical significance (*P* > 0.05).

TIR, time in range; GRI, glycemia risk index; CV, coefficient of variation; MAGE, mean amplitude of glycemic excursion; MMSE, Mini-Mental State Examination; MoCA, Montreal Cognitive Assessment; DST, Digit Span Test; TMT, Trail Making Test; BNT, Boston Naming Test; CDT, Clock Drawing Test; VFT, Verbal Fluency Test.

**Table S3** Neuropsychological test information by tertiles of TIR

| **Variable** | **TIR < 57%**  **(*n* = 32)** | **57% ≤ TIR < 79%**  **(*n* = 32)** | **TIR ≥ 79%**  **(*n* = 32)** | ***P* values** |
| --- | --- | --- | --- | --- |
| TBR population, n (%) | 6 (18.75) | 19 (59.38) | 19 (59.38) | 0.001 |
| MMSE | 28.00 (27.00, 29.00) | 27.00 (26.00, 28.75) | 28.00 (27.00, 29.00) | 0.062 |
| MoCA | 23.00 (22.00, 26.00) | 23.00 (20.00, 24.75） | 23.50 (21.00, 25.00) | 0.471 |
| Immediate memory total score | 24.00 ± 5.20 | 21.91 ± 4.50 | 23.50 ± 5.59 | 0.237 |
| 20-min delayed recall | 7.75 ± 2.50 | 8.00 ± 2.76 | 8.28 ± 2.75 | 0.730 |
| Cued recall | 10.00 (8.25, 11.00) | 10.00 (8.00, 11.75) | 11.00 (9.00, 11.00) | 0.731 |
| Long delayed recognition | 12.00 (10.00, 13.00) | 12.00 (10.00, 14.00) | 12.00 (11.00, 13.00) | 0.867 |
| DST forward | 7.00 (6.00, 8.00) | 6.50 (6.00, 8.00) | 7.00 (6.00, 8.00) | 0.656 |
| DST backward | 4.00 (4.00, 5.00) | 4.00 (3.00, 5.00） | 4.00 (3.25, 5.00) | 0.572 |
| TMTA | 40.90 (32.90, 60.25) | 68.82 (39.00, 150.00) | 39.61 (31.39, 140.15) | 0.052 |
| TMTB | 84.00 (58.44, 300.00) | 300.00 (76.73, 300.00) | 217.00 (47.39, 300.00) | 0.099 |
| BNT | 24.00 (22.25, 27.00) | 23.00 (19.25, 25.00) | 25.00 (22.25, 27.75) | 0.020 |
| CDT | 2.00 (2.00, 3.00) | 3.00 (2.00, 3.00) | 2.00 (2.00, 3.00） | 0.509 |
| VFT | 18.00 (16.00, 21.00） | 16.00 (14.00, 18.00) | 17.50 (14.00, 22.00） | 0.108 |
| Depression scale | 52.50 (47.50, 57.50） | 50.00 (47.50, 55.00） | 53.75 (47.50, 57.43) | 0.561 |
| Negative emotion | 83.00 (74.00, 91.75) | 90.00 (77.00, 97.00) | 87.00 (80.00, 91.00) | 0.329 |

Data are presented as means ± standard deviations or medians (interquartile ranges) for continuous variables, and numbers (percentages) for categorical variables. A two-tailed value of *P* < 0.05 was considered as statistically significant.

MMSE, Mini-Mental State Examination; MoCA, Montreal Cognitive Assessment; DST, Digit Span Test; TMT, Trail Making Test; BNT, Boston Naming Test; CDT, Clock Drawing Test; VFT, Verbal Fluency Test; TIR, time in range; TBR, time below range.
